# Digital manikins to self‐report pain on a smartphone: A systematic review of mobile apps

**DOI:** 10.1002/ejp.1688

**Published:** 2020-11-13

**Authors:** Syed Mustafa Ali, Wei J. Lau, John McBeth, William G. Dixon, Sabine N. van der Veer

**Affiliations:** ^1^ Centre for Epidemiology Versus Arthritis University of Manchester Manchester UK; ^2^ Centre for Health Informatics Division of Informatics, Imaging and Data Sciences University of Manchester Manchester UK; ^3^ NIHR Manchester Musculoskeletal Biomedical Research Centre Central Manchester University Hospitals NHS Foundation Trust Manchester UK; ^4^ Manchester Academic Health Science Centre (MAHSC) University of Manchester Manchester UK

## Abstract

**Background:**

Chronic pain is the leading cause of disability. Improving our understanding of pain occurrence and treatment effectiveness requires robust methods to measure pain at scale. Smartphone‐based pain manikins are human‐shaped figures to self‐report location‐specific aspects of pain on people's personal mobile devices.

**Methods:**

We searched the main app stores to explore the current state of smartphone‐based pain manikins and to formulate recommendations to guide their development in the future.

**Results:**

The search yielded 3,938 apps. Twenty‐eight incorporated a pain manikin and were included in the analysis. For all apps, it was unclear whether they had been tested and had end‐user involvement in the development. Pain intensity and quality could be recorded in 28 and 13 apps, respectively, but this was location specific in only 11 and 4. Most manikins had two or more views (*n* = 21) and enabled users to shade or select body areas to record pain location (*n* = 17). Seven apps allowed personalising the manikin appearance. Twelve apps calculated at least one metric to summarise manikin reports quantitatively. Twenty‐two apps had an archive of historical manikin reports; only eight offered feedback summarising manikin reports over time.

**Conclusions:**

Several publically available apps incorporated a manikin for pain reporting, but only few enabled recording of location‐specific pain aspects, calculating manikin‐derived quantitative scores, or generating summary feedback. For smartphone‐based manikins to become adopted more widely, future developments should harness manikins’ digital nature and include robust validation studies. Involving end users in the development may increase manikins’ acceptability as a tool to self‐report pain.

**Significance:**

This review identified and characterised 28 smartphone apps that included a pain manikin (i.e. pain drawings) as a novel approach to measure pain in large populations. Only few enabled recording of location‐specific pain aspects, calculating quantitative scores based on manikin reports, or generating manikin feedback. For smartphone‐based manikins to become adopted more widely, future studies should harness the digital nature of manikins, and establish the measurement properties of manikins. Furthermore, we believe that involving end users in the development process will increase acceptability of manikins as a tool for self‐reporting pain.

## 1 INTRODUCTION

Chronic pain is a common cause of disability worldwide (Vos et al., 2012) with significant economic costs for society (Breivik et al., 2013). For example, in the United Kingdom, an estimated 28 million adults live with chronic pain (Fayaz et al., 2016), many of them with pain in multiple sites (Carnes et al., 2007). In addition to its high prevalence, chronic pain also has a substantial impact on the physical well‐being and mental health of those suffering from it (Breivik et al., 2006; Hadi et al., 2019; Tsang et al., 2008), which in turn negatively affects other outcomes (Lacey et al., 2015; Smith et al., 2018).

The clinical and self‐management of chronic pain is suboptimal (Mills et al., 2016), and there is a recognised need for epidemiological studies in order to improve this (Dorner, 2018). However, the complex and fluctuating nature of pain (Diatchenko et al., 2013); the fact that people from different backgrounds may express and report their pain differently (Campbell & Edwards, 2012; Krupić et al., 2019); and a lack of high‐quality population studies (Fayaz et al., 2016) hampers our understanding of the pain occurrence and progression and pain mechanisms, as well as the development of effective therapeutical interventions.

Addressing these challenges requires reliable and valid measurement methods (Erdek & Pronovost, 2004; Fayaz et al., 2016) that can be used longitudinally. Currently, there are a range of instruments available to measure pain, ranging from single‐item measures of pain location or overall pain intensity (e.g. Visual Analogue Scale) to multi‐item questionnaires covering several pain domains (e.g. the McGill Pain Questionnaire) (McCormick & Frampton, 2019; Pogatzki‐Zahn et al., 2019). However, whereas single items tend to oversimplify the pain experience (Younger et al., 2009), more complex instruments often require high levels of literacy and engagement (Wood, 2004).

Pain manikins, also known as pain drawings or pain diagrams, are human‐shaped figures where people can shade areas to self‐report the location of their pain more accurately (Jang et al., 2014). Owing to their reliability (Southerst et al., 2013), pain manikins have been used widely to assess pain and support better chronic pain management (Baeyer et al., 2011; Cruder et al., 2018; Grunnesjö et al., 2006). A recent systematic literature review by Shaballout and colleagues provided a comprehensive overview of methodological milestones in the development of pain manikins to date. They showed that paper‐based manikins first appeared in 1949, with a version for personal and tablet computers following 40 years later (Shaballout et al., 2019).

Due to their high uptake and integration into people's daily life, smartphones are an increasingly common technology for collecting self‐reported health data for research (Hicks et al., 2019), self‐management (Najm et al., 2019) and clinical care (Rowland et al., 2020). Compared to their currently available pc and tablet‐based counterparts, smartphone‐based pain manikins require a novel approach to allow for smaller and non‐standardised screen sizes. A smartphone‐based pain manikin opens up the possibility of collecting manikin reports at scale and pace via personal mobile devices. The 2019 app review by Zhao *et al*. reported that 11 of the 36 included pain management apps incorporated some kind of body map or manikin (Zhao et al., 2019). Yet, Shaballout *et al*. did not identify the use of smartphones for pain manikin reports as a methodological milestone. Furthermore, Zhao's review did not provide much detail on the features of the body map functionality, leaving it largely unknown to what extent smartphone‐based manikins are available for measuring pain.

This review, therefore, aimed to gain insight into the current state of play for smartphone‐based pain manikins and formulate recommendations to guide future developments. Specific objectives were to (a) Identify and characterise apps that include a pain manikin; and (b) Within these apps, describe the features of the manikin functionality. We addressed these objectives through a systematic review of app stores. We expect our review to contribute to the development and adoption of manikins for improved pain measurement.

## 2 METHODS

We used the Preferred Reporting Items for Systematic Reviews and Meta‐Analyses (PRISMA) guidelines (Moher et al., 2009) to report the results of our app review process, where relevant.

### 2.1 Search strategy

We searched Apple's App store (UK) and the Google Play store in April 2019 using a two‐step search strategy. First, we performed four separate searches using ‘pain’, ‘pain tracker’, ‘pain diary’ and ‘pain diagram’ as search terms. From these initial searches, we identified painful conditions for which apps were frequently available, such as rheumatoid arthritis, migraine and headache. In the second step, we then used these painful conditions as search terms in combination with the terms from the first search.

To ensure the comprehensiveness of our results, we complemented our search strategy by hand‐searching apps included in reviews published in the last 5 years that focused on pain management (Devan et al., 2019; Lalloo et al., 2017; Zhao et al., 2019).

### 2.2 Inclusion criteria

Apps were eligible for inclusion if they:

- Included a manikin that allowed people to self‐report their pain. For the purpose of this review, we defined a manikin as “a line drawn to outline the human body and to allow reporting of pain in any particular part or location of the body” (Schierhout & Myers, 1996), while also including three‐dimensional body shapes. We excluded non‐interactive manikins, i.e. manikins that did not allow users to manipulate or draw on the body shape (e.g. manikins included as an illustration for educational purposes);
- Had people affected by pain as their target users; apps aimed primarily at healthcare professionals were excluded;
- Had a title and description in English;
- Were available in the United Kingdom;
- Published at any date.

### 2.3 App selection process

After de‐duplicating the results from the searches, two reviewers (SMA, WJL) independently screened all apps for relevance using the criteria as stated above, based on the title and description as provided in the app store. All apps (both free and paid) deemed relevant were downloaded for full review to confirm eligibility; for apps that were excluded at this stage we recorded the reason why. Disagreements about inclusion between reviewers were resolved through discussion, involving a third reviewer (SNVDV) where needed.

### 2.4 Data extraction and synthesis

Each included app was downloaded on a Samsung Galaxy S6 (Android Version 7.0) or iPhone X (IOS 11.2.1) for duplicate and independent data extraction by two reviewers (SMA, WJL). We developed a data extraction form in Microsoft Excel, informed by the published manikin literature (Barbero et al., 2015; Bryner, 1994; Cruder et al., 2018; Hüllemann et al., 2017; Jamison et al., 2004; Leoni et al., 2017; Neubert et al., 2018; Shaballout et al., 2019; Southerst et al., 2013; Stinson et al., 2014; Wenngren & Stålnacke, 2009; Zhao et al., 2019), the Mobile Application Rating Scale (MARS) (Stoyanov et al., 2015) and work from the Initiative on Methods, Measurement, and Pain Assessment in Clinical Trials (IMMPACT) (Dworkin et al., 2005). The research team piloted the data extraction form for completeness and clarity. The final version included the following categories of items (see Tables S1 and S2 in Supplement for the complete list of items and their descriptions):

- **General app characteristics**, including: app name; developer; geographical region; availability (App store; Google Play; both); affiliation (i.e. professional or professional organisations/associations involved in app development yes; no); if end users had been involved in the development process (yes; no/unknown); purchase costs (free; in‐app purchases; costs at download); and log‐in requirements (yes; no);
- **App quality,** using MARS (Stoyanov et al., 2015). MARS consists of 19 items that are rated on a 5‐point scale (1 = Inadequate, 2 = Poor, 3 = Acceptable, 4 = Good and 5 = Excellent). Items are grouped into four dimensions: engagement (e.g. interactivity – does it allow user input, provide feedback and contain prompts?); functionality (e.g. ease of use – how easy is it to learn how to use the app?); aesthetics (e.g. visual appeal – how good does the app look?) and information (e.g. credibility – does the app come from a legitimate source?). In line with previous studies (Devan et al., 2019; Salazar et al., 2018; Stoyanov et al., 2015), two reviewers independently rated for each app all MARS items and calculated mean scores per dimension and the mean overall quality score across dimensions. Discrepancies in dimension and overall scores between reviewers were resolved by taking the mean. To further ensure a shared understanding of MARS items between reviewers, we provided additional information to illustrate how to apply each item to the pain manikin apps in our review. For the MARS item “Has the app been trialled and tested,” we used the app name to search Google Scholar for empirical studies evaluating the usability, effectiveness or other aspects of the app.
- **Recording of pain intensity and quality**; this category referred whether users could record pain intensity and pain quality in the app, and to what extent it was possible to enter different values for different locations. Pain intensity and pain quality are recommended as essential measures within the core outcome domain of ‘pain’, which is one of the six domain to be considered when designing chronic pain clinical trials, as recommended by the Initiative on Methods, Measurements, and Pain Assessment in Clinical Trials (IMMPACT) (Dworkin et al., 2005). IMMPACT describes pain intensity as the overall magnitude of the pain, while referring to pain quality as capturing its sensory and affective characteristics. We considered other pain aspects recommended by IMMPACT (e.g. rescue treatment) out of scope for this review because they were less likely to be location specific.
- **Manikin features**, including items related to: layout (i.e. the look of the manikin; e.g. level of detail of the body figure); interaction (i.e. the feel of the manikin; e.g. method for recording pain location in the manikin) and any other input directly related to manikin (e.g. free‐text field qualitatively describing the manikin report) (see Table S1 for additional detail on individual items)
- **Summaries and feedback of manikin reports;** this referred to if and how manikin reports were summarised (i.e. manikin metrics, e.g. number of painful areas, mean pain intensity score across locations); how manikin reports and summaries were presented back to users; and whether users could share reports and summaries with others.

Reviewers met regularly throughout the data extraction process to develop a common understanding of items and solve any discrepancies, involving a third reviewer (SNVDV) if needed. We thematically analysed and synthesised the extracted data in line with our study objectives, using descriptive summary statistics where relevant.

## 3 RESULTS

**Figure 1:**
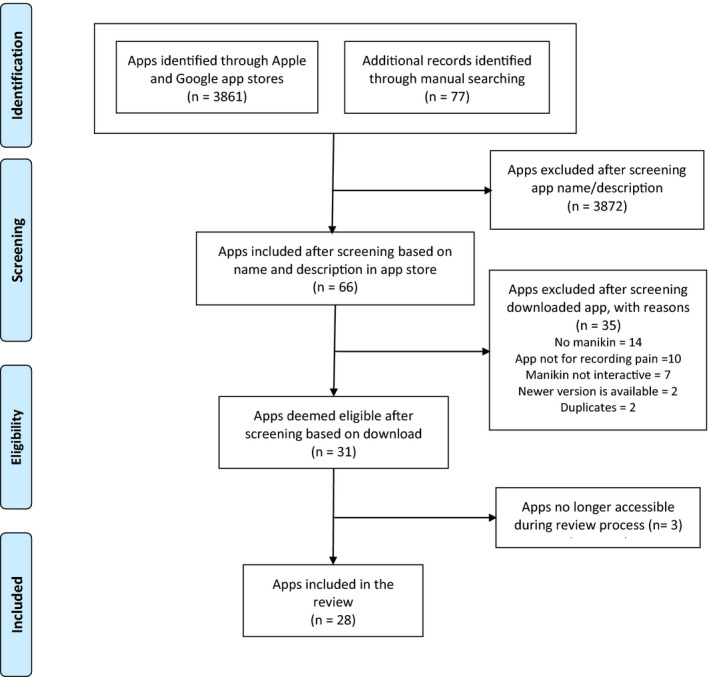
flow diagram of the app selection process

Figure 1 shows how our search yielded 3,938 apps, of which we deemed 31 eligible for inclusion in our review. However, since three apps became unavailable during the review process, we finally included 28 apps for data extraction and synthesis.

Table 1 displays the general characteristics of included apps. More than half of them (*n* = 15) were developed in North America, and 12 were available in the Apple app store either exclusively or together with Google App store. The majority of apps were available for download at no cost (*n* = 24), with four offering additional functionality after in‐app purchases. Of the four requiring payment to download, costs ranged from US$ 0.99 to 6.99. Nine apps mentioned the input from healthcare professionals or professional associations (e.g. the International Children's Palliative Care Network, Society of Critical Care Medicine) in the app description. However, none of the app descriptions mentioned the involvement of end users in the process of app development, leaving this aspect unknown.

**TABLE 1:** General characteristics and quality assessment scores of included apps (*n* = 28) (values are numbers (%) unless indicated otherwise)

*Characteristic*	*Number (%)*
General characteristics	
Geographical region	
North America	15 (54)
Europe	8 (29)
Asia	2 (7)
Africa	2 (7)
Australia	1 (3)
Availability	
Apple App store only	12 (43)
Google Play only	6 (21)
Both	10 (36)
Affiliation^a^	
Yes	9 (32)
No	19 (68)
Purchase costs	
Free to download	20 (72)
Free to download with in‐app purchases	4 (14)
Costs to download	4 (14)
Login requirements	
Yes	13 (46)
No	15 (54)
App quality (MARS scores)^b^	Mean (*SD*)
Overall quality score	3.3 (0.8)
Engagement dimension score	3.0 (0.9)
Functionality dimension score	3.8 (0.8)
Aesthetic dimension score	3.3 (0.9)
Information quality dimension score	2.9 (0.8)

Abbreviations: MARS: Mobile App Rating Scale; *SD*: Standard Deviation.

^a^ Refers to mention of involvement of individual health professionals and/or professional institution/association in the app description

^b^ Scores can range from 1 (inadequate) to 5 (excellent).

### 3.1 App quality

Table 1 shows that, on a scale of 1 to 5 (with higher scores indicating better quality), the mean overall quality score across apps was 3.3 (*SD* = 0.8); the best‐ and worst‐rated app scoring 4.5 and 2.1 respectively. Mean scores for the engagement, functionality, aesthetics and information dimensions were 3.0 (standard deviation [*SD*], 0.9), 3.8 (*SD*, 0.8), 3.3 (*SD*, 0.9) and 2.9 (*SD*, 0.8), respectively (see supplementary Table S3 for app‐specific scores). Most apps (*n* = 21) scored highest on the functionality dimension and lowest on the information dimension (*n* = 18). Based on the app description and our Google Scholar search, none of the assessed apps appeared to have been trialled or tested (e.g. for usability, reliability, effectiveness).

### 3.2 Recording of pain intensity and pain quality

Table 2 shows that pain intensity could be recorded in all 28 apps, but only in 11 could users enter different intensity scores for different locations. Thirteen apps enabled recording of pain quality, with four giving the option of location‐specific values. Examples of available pain quality descriptors included ‘stabbing’, ‘burning’, ‘throbbing’ and ‘shooting’. The four apps with location‐specific pain quality also enabled recording of location‐specific intensity.

**TABLE 2:** Overview of which apps enabled self‐reporting of pain intensity and pain quality^a^, and which of those allowed recording of location‐specific pain intensity and pain quality

App name ↓	Pain intensity		Pain quality	
	Self‐report enabled	Location specific	Self‐report enabled	Location specific
Chronic Pain tracker	Yes	Yes	Yes	‐
Feel My Pain	Yes	Yes	Yes	Yes
GeoPain @Home	Yes	Yes	‐	‐
iMigraine – Migraine Tracker	Yes	‐	‐	‐
Joint Pain Tracker	Yes	‐	‐	‐
Mend+	Yes	Yes	‐	‐
Migraine Buddy – The Migraine and Headache Tracker	Yes	‐	‐	‐
Migraine Headache Diary Head App	Yes	‐	Yes	‐
Migraine Monitor	Yes	‐	‐	‐
My Pain Diary: GOLD EDITION	Yes	‐	Yes	‐
myVectra	Yes	Yes	‐	‐
Ouchie: a pain management companion	Yes	‐	Yes	‐
Pain Assessment Tool for Children	Yes	‐	Yes	Yes
Pain Cal	Yes	‐	Yes	‐
Pain Companion	Yes	Yes	‐	‐
Pain Diary (Privacy Friendly)	Yes	‐	Yes	‐
Pain Diary & Forum CatchMyPain	Yes	Yes	Yes	‐
Pain & Opioid Safety	Yes	Yes	Yes	‐
Pain Scored	Yes	‐	Yes	‐
Pain Tracker & Diary	Yes	Yes	Yes	Yes
Pain Tracker HD	Yes	‐	‐	‐
Pain Tracker	Yes	‐	‐	‐
Patient Communicator	Yes	Yes	Yes	Yes
RA Monitor	Yes	‐	‐	‐
RAPA – RA Patient Application	Yes	‐	‐	‐
RheumBuddy	Yes	‐	‐	‐
Simple Pain Scale	Yes	‐	‐	‐
The O Lab Pain Tracking	Yes	Yes	‐	‐

^a^ IMMPACT (Dworkin et al., 2005) describes pain intensity as the overall magnitude of the pain, and pain quality as the different sensory and affective characteristics of pain.

### 3.3 Manikin features

**Figure 2:**
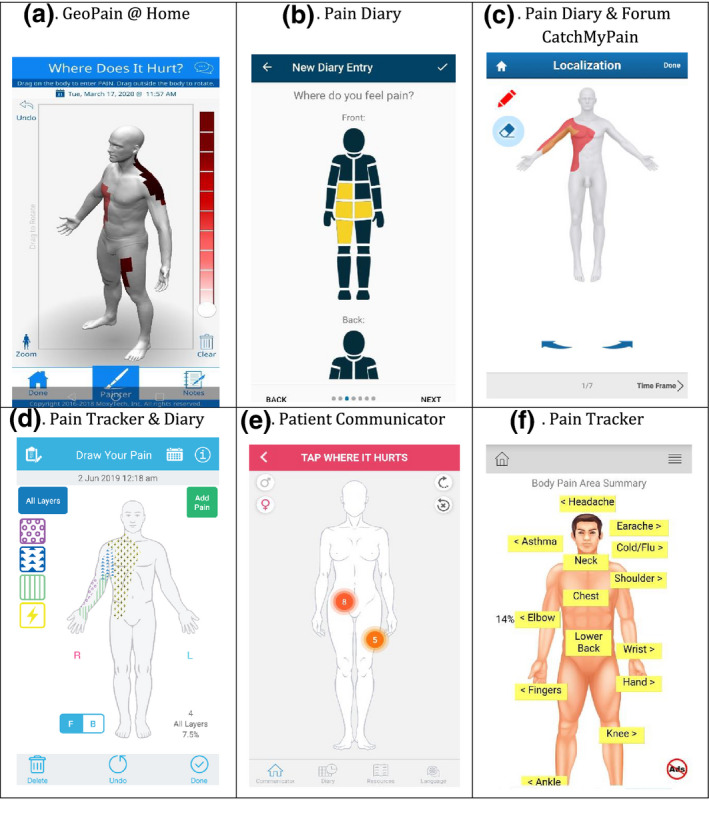
Examples of manikins in included apps to illustrate the variety of manikin features and how we assessed them. GeoPain @Home (a): fully rotatable 3‐D manikin with highly detailed body anchors; zoom and undo options; user can add free‐text description to manikin report. Pain Diary (b): 2‐D manikin with two views, front‐back labels and no body anchors; users can tap on manikin to select segments with only one level of pain intensity. Pain Diary &amp; Forum CatchMyPain (c): 3‐D manikin, offering four views, with high level of body anchors; users can shade areas by drawing to record location‐specific pain intensity. Pain Tracker &amp; Diary (d): two‐dimensional manikin, with gender personalisation and left/right labels; moderate level of body anchors; users can shade areas by drawing on manikin with location‐specific pain intensity and quality (shown as layers); zoom and undo options; users can add free‐text description to manikin reports. Patient Communicator (e): 3‐D manikin, offering two views, with high level of anchor detail and gender personalisation. Users can tap on manikin to mark pain location with a circle and can assign location‐specific pain intensity score and can chose pain quality from a pre‐defined list; a number represents location‐specific intensity; undo option. Pain Tracker (f): 3‐D manikin with 1 view and a high level of anchor detail; users can select a location from a drop‐down list with pre‐defined locations or tapping the label for that location on the manikin; no recording of location‐specific pain aspects

Table 3 shows that the majority of manikins: had more than one view (*n* = 21; e.g. front and back); used labels to support orientation (*n* = 15; mostly ‘left’ and ‘right’) and represented the body figure two dimensionally (*n* = 19) with at least moderately detailed body anchors (*n* = 16). Figure 2 displays six examples of included pain manikins to illustrate the broad range of manikin features and how we assessed them.

**TABLE 3:** Summary of manikin features

*Manikin features*	*Number (%)*
Number of views	
One	7 (25)
Two	15 (54)
More than two	6 (21)
Labels for orientation	
Yes	15 (54)
No	13 (46)
Number of dimensions	
Two	19 (68)
Three	9 (32)
Level of detail of body anchors^a^	
Low	11 (39)
Moderate	9 (32)
High	8 (29)
Zoom option	
Yes	13 (46)
No	15 (54)
Manikin personalisation options	
Yes	7 (25)
No	21 (75)
Free‐text field directly related to manikin report	
Yes	5 (18)
No	23 (82)
Method to record pain location	
Shading any area by drawing on manikin^b^	8 (28)
Shading pre‐defined areas by tapping on manikin^c^	9 (32)
Marking area by tapping on manikin^d^	10 (36)
Selecting locations from pre‐defined list/labels^e^	1 (4)
Option to undo last drawing action	
Yes	23 (82)
No	5 (18)

^a^ For example, anchors indicating clavicle, abdominal muscles, scapulae, spine, hips, thighs, knees, ankle and groin.

^b^ See panels a, c, d in Figure 2

^c^ See panel b in Figure 2

^d^ Refers to apps where users could tap on the body figure to indicate location, after which a mark appeared (e.g. cross, dot or label). See panel e in Figure 2

^e^ See panel f in Figure 2

In 13 apps, users could zoom in on specific areas of the manikin. The *GeoPain @Home* app (see Figure 2, panel a) had a fully rotatable manikin, offering users an infinite number of views. Some apps (*n* = 7) allowed personalisation of the body diagram. For example, in *Pain Assessment Tool for Children* app, users could select the gender, age and skin colour of the manikin. Moreover, in five apps, users could add free‐text notes directly related to their manikin report.

The most common method that apps offered for indicating pain location was by tapping on predefined segments on the manikin in order to shade them (*n* = 13; e.g. Figure 2, panel b). In more than one quarter of the apps (*n* = 8), users could shade any painful area by drawing directly on the manikin (e.g. Figure 2, panel a, c, d), while the remaining apps used simpler methods, including marking a location with a cross, dot or other label by tapping on manikin (*n* = 5; e.g. Figure 2, panel e), and selecting pre‐defined labels on the manikin or from a list (*n* = 2; e.g. Figure 2, panel f).

### 3.4 Summaries and feedback of manikin reports

Table 4 shows that a total of 12 apps calculated 9 different manikin metrics to summarise manikin reports. The most common metrics were related to location (*n* = 6), such as the frequency with which a body manikin area was affected in a specified time period. Five apps calculated metrics related to pain extent. Of those, only three calculated the % of body area affected, despite eight apps having the theoretical possibility to provide this metric (i.e. because users could shade any painful area directly on the manikin). Of the 14 apps that enabled users to record their location‐specific pain intensity, four provided mean intensity as a metric. Only two apps combined multiple pain aspects into a single, composite metric: *GeoPain @Home* and *Feel my Pain* calculated pain scores by combining pain extent and intensity, but without specifying the score's underlying formula.

**TABLE 4:** Overview quantitative manikin metrics that apps provided to summarise manikin reports; in total, 12 apps provided at least one manikin metric

Manikin metric	Metric definition	Apps that calculated the metric
Pain extent (*n* = 5)		
% body manikin area affected	% of the total manikin that had been shaded with any intensity	GeoPain @ Home^a^; Pain Tracker & Diary; Pain Diary & Forum CatchMyPain
Number of affected body manikin areas	Total number of pre‐defined body manikin segments or joints that had been selected with any intensity	Pain Scored; Pain Diary (Privacy Friendly)
Pain location (*n* = 6)		
Body manikin area affected in a specified time period	Whether a pre‐defined body manikin segment or joint had been selected with any intensity yes/no in a time period	myVectra; RAPA – RA Patient Application
Frequency with which a body manikin area was affected in a specified time period	Number of times a pre‐defined body manikin segment or joint had been selected with any intensity in a time period	Pain Scored^d^; Migraine Headache Diary HeadApp; RheumaBuddy;
Number of symptoms per affected body manikin area	Number of symptoms that users had selected for a pre‐defined body manikin segment	RA Monitor;
Pain intensity (*n* = 4)		
Mean intensity across the body manikin	Weighted mean pain intensity across all shaded areas on the manikin^b^	GeoPain @ Home^a^; Pain Tracker & Diary;
Mean intensity per affected body manikin area	Mean pain intensity for a specific body manikin area or pre‐defined segment	GeoPain @ Home^a^, ^c^; Pain Diary & Forum CatchMyPain; The O Lab Pain Tracking
Composite metrics (*n* = 2)		
P.A.I.N.S. score	Combines mean intensity and % body area affected, but the underlying formula is not specified	GeoPain @Home^a^
Pain score^e^	Not provided	Feel my Pain

^a^ GeoPain @Home provided all metrics at the level of the full body manikin, as well as for 14 pre‐defined body parts (e.g. mouth, right hand, pelvis, etc.)

^b^ We assumed that apps calculated a weighted mean intensity, but only *Pain Tracker & Diary* explicitly stated this.

^c^ Displayed as colours in a summary manikin; colours referred to the original pain intensity scale of 0–10

^d^ Displayed as colours in a summary manikin; colours referred to three categories (low (<−1σ), medium (σ) and high (>1σ)). No explanation of what σ means in this context.

^e^ Presented as ‘'Your score x out of 10', with higher values reflecting a better state

**Figure 3:**
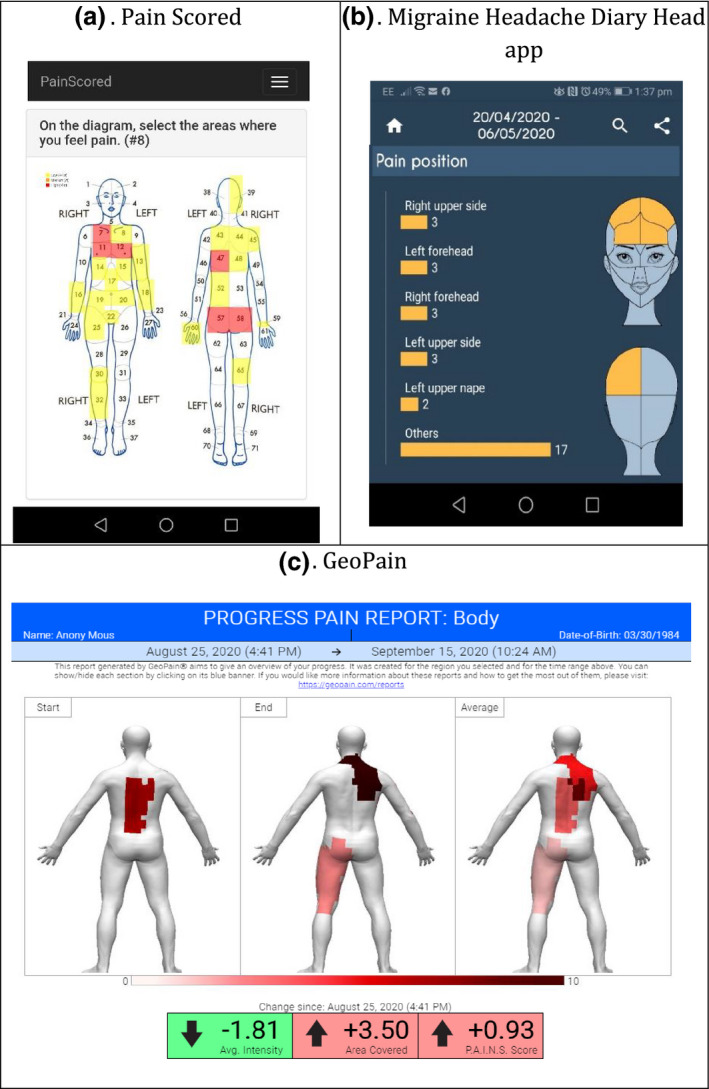
Examples of different types of manikin feedback. Pain Scored (a): body segments are shaded based on the selection of a body segment at least once over a specified time period. Migraine Headache Diary Head app (b): single metric over time, presented as a graph. Geo Pain (c): mean location‐specific intensity, presented as a heat map

The majority of apps (*n* = 22) had an archive function in the form of a list, calendar or graph where users could access historical, individual entries. For the majority of these (*n* = 15), entry‐level feedback included an image of the manikin report. In four apps, users could easily browse through the archive of images (e.g. using a slider) for a ‘pain time‐lapse’ to see how their pain had changed over a certain period. Only some apps (*n* = 8) provided feedback in the form of a summary manikin that combined pain entries across manikin reports: for example, by shading all body segments that had been selected at least once over a specified time period (see Figure 3, panel a), or by creating a ‘heat map’ to feed back the mean location‐specific intensity (see Figure 3, panel c). Of the 12 apps that calculated at least one manikin metric, five provided users with a graph of that metric over time (see Figure 3, panel b). If fed back numerically, metric values were presented at the level of one manikin report (*n* = 6), across reports in a specified time period (*n* = 8), or both (*n* = 1). *Geopain @Home* also presented a change in metric value between the first and last manikin report in the selected time period.

Eleven apps enabled users to share their manikin report with others via email, e.g. by creating a PDF, or a URL to access the report directly. Among these 11 apps, *Ouchie* also allowed users to share their reports with an in‐app community to facilitate discussion and peer support.

## 4 DISCUSSION

This review included 28 smartphone apps that were publically available in app stores in the United Kingdom and that incorporated a manikin for pain reporting. The mean overall quality score of apps was 3.3 of 5. For all of them, it was unclear if they had been trialled or tested, and whether they had involved end users in the development. All apps enabled users to record pain intensity, but this was location specific in less than half of them. The majority of manikins had at least two or more views presented two dimensionally and with at least moderately detailed body anchors, where users could select pre‐defined body segments to indicate the location of their pain. One in four apps offered the option of personalising the manikin appearance. Almost one in two apps calculated at least one metric to quantitatively summarise manikin reports, with location being most common. Although almost all apps had an archive function where users could access historical manikin reports, only few offered feedback that summarised manikin reports over time.

### 4.1 Relation to other studies

We identified 31 apps that incorporated a pain manikin, of which we included 28 in our review. This number is similar to or higher than other recent reviews of pain management apps, which identified 36 (of which 11 included a manikin) (Zhao et al., 2019), 19 (Devan et al., 2019) and 10 (Lalloo et al., 2017). All expressed concerns about ad hoc (Zhao et al., 2019) or no engagement (Devan et al., 2019; Lalloo et al., 2017) of end users in the process of app development; our findings seem to support these concerns.

Only a few apps in our review offered personalisation of manikin features. This finding aligns with that of Devan et al, who reported that none of the self‐management apps included culturally tailored aspects (Devan et al., 2019). A meta‐ethnographic review exploring patients’ perceptions, beliefs and experience of mHealth apps highlighted the lack of personalisation as one of the major concerns (Vo et al., 2019).

We found that 11 of 28 apps enabled users to report location‐specific pain aspects in the manikin, which is a similar proportion identified by Zhao et al. (2019), who reported this for 4 of 11 apps included in their review.

Our results also showed that none of the apps, or the manikins within them, were trialled or tested. This hampers their use in clinical or research settings, which is different for paper‐based manikins, which have been evaluated more extensively (Chatterton et al., 2013; Southerst, et al., 2013). Zhao et al. and Devan et al. similarly concluded that the pain management apps in their review were not suitable for clinical usage, mainly due lack of: patient and clinician involvement in the development process; support for patients to communicate with clinicians and evaluations of effectiveness (Devan et al., 2019; Zhao et al., 2019).

### 4.2 Implications for future development of smartphone‐based pain manikins

#### 4.2.1 Developers should harness the digital nature of manikins

For smartphone‐based manikins to be adopted more widely, they need to offer clear advantages compared to their well‐established, paper‐based counterparts (O’Donnell & Curley, 1985; Öhlund et al., 1996). Their digital nature has several potential benefits, including: easy, longitudinal recording of location and location‐specific aspects of pain; more realistic and dynamic features (e.g. a three‐dimensional manikin that users can view from any angle); quick and reliable processing of manikin reports into quantitative pain scores (i.e. manikin metrics), established (e.g. % body area affected) as well as novel ones (e.g. composite metric combining pain extent and intensity); and instant and customisable feedback of manikin reports over time. However, our review shows that these benefits have not yet fully materialised, with most manikins offering relatively basic functionality. This suggests there is ample opportunity for developers to better harness the digital nature of future smartphone‐based manikins, and for researchers to investigate the effect of these enhancements on users’ engagement with manikins over time.

### 4.3 Researchers should establish the measurement properties of manikins

Confirming the robustness of smartphone‐based manikins as a pain measurement instrument is another prerequisite for these tools to be adopted more widely. In keeping with previous reviews (De La Vega & Miró, 2014; Haskins et al., 2017), our review suggests that there is a gap between apps that are publically available and those that have been investigated in research studies. There have been studies on the reliability of pain manikins developed for larger mobile devices (e.g. tablets) (Barbero et al., 2015; Leoni et al., 2017; Neubert et al., 2018), but those findings do not necessarily translate to manikins developed for smaller, non‐standardised smartphone screens. Future research should, therefore, focus on assessing the measurement properties (e.g. reliability, validity, responsiveness) of smartphone‐based pain manikins in line with international guidance (Mokkink et al., 2010), in order for them to become an accepted standard for measuring location‐specific pain aspects in trials and epidemiological studies. This will also contribute to embedding these manikins into interventions aimed at improving clinical and self‐management of painful conditions.

### 4.4 Developers should consider involving representative end user groups in the development process

Involving end users will ensure that apps incorporating a manikin can reach their intended objectives (McCurdie et al., 2012). Furthermore, we believe that end user involvement will increase the chance of manikin reports being an adequate reflection of people's pain, thereby increasing their acceptability as a self‐reporting tool. Pain prevalence is highest among black and Asian minority ethnicities (Macfarlane et al., 2015), who may experience or report pain differently compared to Caucasians (Mills et al., 2019). The same has been suggested for other groups, such as older people, or those suffering from multisite pain (Mills et al., 2019). Therefore, involving representative end user groups may ultimately contribute to better content and cross‐cultural validity of manikins as measurement instruments (Mokkink et al., 2010), and enhance the usability and usefulness of manikins for a broad range of people living with a painful condition. The fact that only few of the apps allowed cultural or other personalisation of the manikin appearance may indicate that there is room for improvement in this area.

### 4.5 Limitations

One limitation of our review is that it was designed to identify manikins that were incorporated within apps that were publically available in UK app stores. This implies that our search strategy did not allow us to identify smartphone‐based manikins offered in app stores outside the United Kingdom, or by healthcare providers or researchers via other routes. However, the literature review by Shaballout *et al*. (Shaballout, et al., 2019) did not identify them as a methodological milestone, suggesting that peer‐reviewed reports on smartphone‐based manikins are scarce at most. This means there is still a gap in knowledge on how screen size and other device characteristics affect user engagement in and measurement properties of smartphone‐based manikins.

Another limitation is that for our data on general app characteristics and parts of the app quality appraisal, we relied on information provided in the app stores, but without contacting the app developers to confirm and complement it. This would have allowed us to better interpret missing information on, for example, end user involvement (i.e. distinguish ‘not reported’ from ‘not undertaken’) and whether apps had been trialled.

## 5 CONCLUSION

This systematic review of app stores identified a substantial number of publically available apps that incorporated a manikin for pain reporting. Although this number is encouraging, only few apps offered functionality to: record location‐specific pain aspects; calculate quantitative scores based on manikin reports or generate engaging manikin summary feedback. This limits their added value compared to their paper‐based counterparts. Future developments of smartphone‐based manikins should better harness the manikins’ digital nature and include robust studies to establish their measurement properties. We also believe that involving end users in the development process will increase acceptability of manikins as a tool for self‐reporting pain. Ultimately, this will lead to smartphone‐based manikins becoming an accepted standard for measuring location‐specific pain aspects for research, self‐management and clinical care.

## CONFLICT OF INTEREST

None of the authors has any conflict of interest.

## DISCLOSURE

This work, either in part or full, is not presented previously.

## AUTHOR CONTRIBUTIONS

SNVDV, WGD and JM conceived the idea of this app review. SMA, WJL and SNVDV extracted and synthesised the data. All authors were involved in interpreting the data. SMA drafted the manuscript. All authors revised it critically for important intellectual content and approved the final version of manuscript for submission. SMA and SNVDV take responsibility for the integrity of the work as a whole.

## Supporting information

Table S1‐S3
