# Supplementary material for: Digital manikins to self‐report pain on a smartphone: A systematic review of mobile apps
Source: Eur J Pain. 2020 Nov 13;25(2):327–38. doi: 10.1002/ejp.1688 (PMC7839759; doi:10.1002/ejp.1688)

# Supplementary material

**Table S1: List of data extraction items and their description**

| ***Item name*** | ***Description*** | ***Can the user record this?*** | ***How can the user record this?*** |
| --- | --- | --- | --- |
| **LAYOUT: Look of the manikin** | | | |
| Level of detail | Are there anchors available to help users to identity correct body location? | 1. Very low (no anchors)  2. Low (basic anchors only)  3. Medium (all main anchors present  4. High (all main anchors + additional details  5. Very high (close to photo quality | Key anchors (clavicle, sternum, abdominal muscles, shoulder blades, hips, thigh, knees, ankle, etc.); labels yes/no; etc. |
| Labels for orientation | Are textual labels provided to aid orientation by users? For example, left and right | 1. Yes  2. No | Label description. If front/back, left/right labels are used |
| **INTERACTION: General feel of the manikin** | | | |
| Zoom option | Can the user zoom in on specific body areas? | 1. Yes  2. No | Pinch, double-tap, single-tap, pre-defined zoomed-in areas; etc |
| Pain location recording - method | How can users the location of their pain on their manikin? | 1. Drawing areas directly on manikin 2. Tapping to mark areas directly on the manikin 3. Selecting segments directly on the manikin 4. Selecting locations from a list 5. Other (please specify) | Additional information on how users interaction if indicating pain. For example:   - if drawing directly, option to select different pen sizes for drawing; etc. - If selecting segments or locations: how many segments; with/without labels (give examples); etc. |
| Pain location recording - display | How is user-recorded pain display on the manikin? | 1. Coloured area or segment 2. Cross/mark on indicated pain location 3. Other (please specify) | Additional info on how pain is displayed. For example, |
| Manikin personalisation | Does manikin allow personalisation by the user? For example, gender, ethnicity, age, body shape, etc. | 1. Yes  2. No | What aspects can be personalised?  How can users personalise the manikin. For example, selecting gender during setting up account gives gender-specific manikin; or separate manikin configuration menu in settings; etc. |
| Function to undo manikin drawing action | Can the user undo or erase any drawing actions on the manikin? | 1. Yes  2. No | Eraser function; undo last action; etc. |
| Number of views | Number of views/sides that user can select | 1. 1-view 2. 2-views 3. 3-views 4. 4-views 5. User can rotate manikin in any position | Additional information on which sides (front, back, left, right; above/below). Specific views for specific sides (e.g. below the feet) |
| Dimensions | Number of dimensions; we consider manikins 3D if they have shades, curves, depth, etc. | 1. 2D  2. 3D | Not applicable |
| **INPUT: What pain-related information the user can record in the manikin and in other parts of the app** | | | |
| Location-specific pain intensity | Can the user record different pain intensity levels for different locations within one manikin report? One score for location of greatest concern is not considered location-specific | 1.Yes  2. No | Numerical, textual, emoticons, colours, etc.  Type of scale; number of points; anchors; content of textual labels  How can users record intensity in the app flow |
| Pain quality | Can the user record their pain quality? Pain quality includes pain type, how the pain feels, etc. For example, stabbing, throbbing, dull, etc. | 1. Yes, location-specific  2. Yes, but not location-specific (i.e. different qualities across different locations)  3. No | Textual, icons, colours, patterns, etc.  Textual labels; types of icons |
| Overall pain intensity | Per manikin report, can the user record their overall pain intensity? | 1.Yes  2. No | How is the question formulated; answering options; at what point in the app flow is the question asked; |
| Free text fields | Are there free text fields available for the user to record comments, diary entries, etc? | 1. Yes  2. No | Label for free text fields; where in the app flow are they; indicate if specifically related to manikin report |
| **OUTPUT: how recorded pain data is presented back to users and if they can share their pain reports** | | | |
| Description of manikin-related feedback | How users are fed back with their self-reported pain? | Not applicable | How is manikin feedback presented; as an archiving feature or as a report? What information, extracted from the manikin report, is provided within archive or report? How self-reported pain is summarised, either graphically or numerically or both? |
| Name of manikin summary metrics | Does the app translate the manikin report into a summary metric? | 1. Pain extent  2. Pain location  3. Pain intensity  4. Composite metrics | What is the name of metrics that can be judged from the manikin report and how it was created; whether app names the summary metrics or not. Name of the apps that have used specific summary metrics. |
| Description (given in app) | What label/name is given within the app to describe a summary metric? | 1. Yes  2. No | How summary metrics are described in the app, retaining same terminologies and meaning?  How that description helps us to relate that metric to common summary metrics across different apps |
| Options to share pain data | Can the user share their pain data with others? | 1.Yes  2.No | What data can they share; with whom?; how?; etc. |

**Table S2: Description of MARS items and additional information for applying them in this systematic review**

| **Domain** | **Item** | **Description and additional information** | |
| --- | --- | --- | --- |
| Engagement | Entertainment | MARS description | Is the app fun/entertaining to use? Does it use any strategies to increase engagement through entertainment (e.g. through gamification)? |
|  |  | Additional information | Mobile application can have fun or game elements despite purpose is to record pain. Game elements promote users' engagement, e.g., leader-board |
|  | Interest | MARS description | Is the app interesting to use? Does it use any strategies to increase engagement by presenting its content in an interesting way? |
|  |  | Additional information | Interest involves the ways with which app features and its content is presented. For example information is presented in the form of plain text, making it less interesting to read |
|  | Customisation | MARS description | Does it provide/retain all necessary settings/preferences for apps features (e.g. sound, content, notifications, etc.)? |
|  |  | Additional information | Does the app provide a setting tool which lets users change the user interface so that it is best suited for them? This can be either related to the pain reporting process or just for the app in general. |
|  | Interactivity | MARS description | Does it allow user input, provide feedback, contain prompts (reminders, sharing options, notifications, etc.)? Note: these functions need to be customisable and not overwhelming in order to be perfect. |
|  |  | Additional information | In addition to MARS description, description of manikin in terms of dimension (2D/3D), orientation (left/right) and gender (male/female/neutral) can be considered as features of interactivity |
|  | Target group | MARS description | Is the app content (visual information, language, design) appropriate for your target audience? |
|  |  | Additional information | Same as MARS |
| Functionality | Performance | MARS description | How accurately/fast the app’s features (functions) and components (buttons/menus) work? |
|  |  | Additional information | To check whether app works slowly, troubles sign-in, takes time in processing data and/or does it crash. (Note that this may be overestimated as apps are assessed on a one-time-use basis) |
|  | Ease of Use | MARS description | How easy is it to learn how to use the app; how clear are the menu labels/icons and instructions? |
|  |  | Additional information | Does the app or app description on play store provide a tutorial (audio-visual or textual)?  Also, are different tabs in the app well-labelled and the terms well-described |
|  | Navigation | MARS description | Is moving between screens logical/accurate/appropriate/ uninterrupted; are all necessary screen links present? |
|  |  | Additional information | Same as MARS. For example, while looking at report, can you go back to data entry for entering new record or editing previously entered record |
|  | Gestural Design | MARS description | Are interactions (taps/swipes/pinches/scrolls) consistent and intuitive across all components/screens? |
|  |  | Additional information | Same as MARS |
| Aesthetics | Layout | MARS description | Is arrangement and size of buttons/icons/menus/content on the screen appropriate or zoomable if needed? |
|  |  | Additional information | Same as MARS. In addition, assess whether reporting through manikin considers different users’ requirement and allows selecting a specific location by zooming. |
|  | Graphics | MARS description | Graphics: How high is the quality/resolution of graphics used for buttons/icons/menus/content? |
|  |  | Additional information | Same as MARS |
|  | Visual Appeal | MARS description | Visual appeal: How good does the app look? |
|  |  | Additional information | Same as MARS |
| Information | Accuracy of App Description | MARS description | Does app contain what is described? |
|  |  | Additional information | Judged based on comparing what is seen in the apps with the description and images provided on the app store |
|  | Goals | MARS description | Does app have specific, measurable and achievable goals (specified in app store description or within the app itself)? |
|  |  | Additional information | Does the app set out objective(s) for users and/or provide in-app feature of goal setting to promote pain management |
|  | Quality of Information | MARS description | Quality of information: Is app content correct, well written, and relevant to the goal/topic of the app? |
|  |  | Additional information | Assess the content, whether it provides factually correct information, if provided at all. Check if graphs and report generated are based on entered data |
|  | Quantity of Information | MARS description | Is the extent coverage within the scope of the app; and comprehensive but concise? |
|  |  | Additional information | Check how comprehensive the app is in terms of features, such as pain recording, report generation, reminder, feedback, social support, sharing report, in-app advice/feedback, history taking, information to read. |
|  | Visual Information | MARS description | Is visual explanation of concepts – through charts/graphs/images/videos, etc. – clear, logical, correct? |
|  |  | Additional information | If app presents key concepts related to pain and its management which are potentially of user's interest and if they are clear and correct |
|  | Credibility | MARS description | Does the app come from a legitimate source (specified in app store description or within the app itself)? |
|  |  | Additional information | In addition to MARS description check for credits and website of app (if available) |
|  | Evidence base | MARS description | Has the app been trialled/tested; must be verified by evidence (in published scientific literature)? |
|  |  | Additional information | Same as MARS (will check in Google Scholar with standard key words, such as name of application, name of developer, pain management app, mHealth app) |

**Table S3: App-specific scores for MARS dimensions, reported per reviewer and as total mean (i.e. mean between reviewers)**


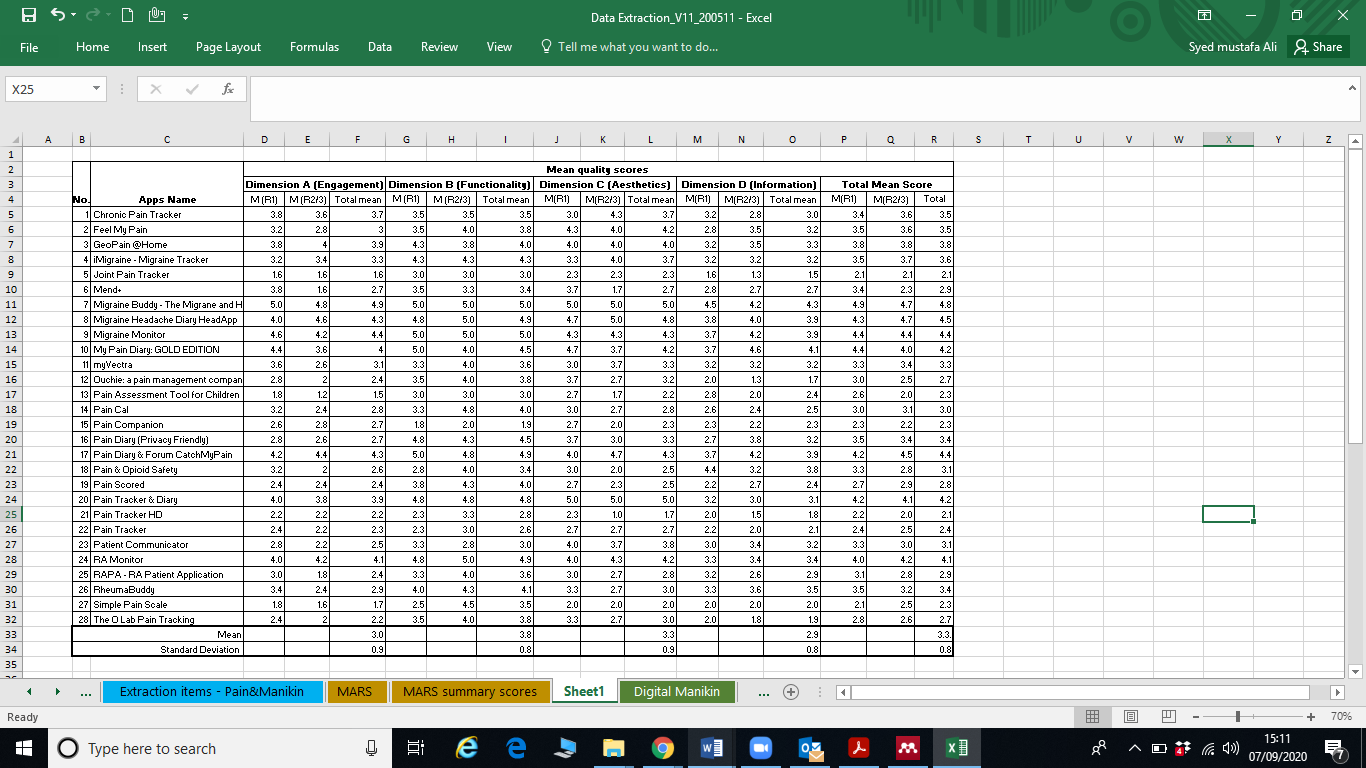

Supplement: Supplementary file 1 — Table S1‐S3 [file EJP-25-327-s001.docx]
